# Acute hypoxia stress mediates *HIF-1α-Yki-Cactus* axis to facilitate the infection of *Vibrio parahaemolyticus* in *Litopenaeus vannamei*

**DOI:** 10.3389/fimmu.2024.1476309

**Published:** 2024-11-27

**Authors:** Honghui He, Shaoqing Huang, Ningze Geng, Shaoping Weng, Jianguo He, Chaozheng Li

**Affiliations:** ^1^ State Key Laboratory of Biocontrol/School of Marine Sciences, Sun Yat-sen University, Guangzhou, China; ^2^ Southern Marine Sciences and Engineering Guangdong Laboratory (Zhuhai), Zhuhai, China; ^3^ School of Life Sciences, Sun Yat-sen University, Guangzhou, China; ^4^ China-ASEAN Belt and Road Joint Laboratory on Mariculture Technology, Guangzhou, China; ^5^ College of Marine Sciences, Beibu Gulf University, Qinzhou, China

**Keywords:** hypoxia, shrimp, disease, HIF-1α-Yki-Cautus, Vibrio parahaemolyticus

## Abstract

**Introduction:**

Hypoxia stress renders aquatic animals more susceptible to bacterial disease, yet the underlying mechanism remains elusive.

**Methods:**

We conducted an acute hypoxia stress experiment to investigate the impact of stress on the immune response of *Litopenaeus vannamei* via transcriptome analysis, RT-qPCR and Western blot.

**Results:**

Our results showed that acute hypoxia stress disrupted the tissue architecture, and significantly changed the gene expression profiles in the hepatopancreas of shrimp. More importantly, acute hypoxia stress significantly changed the expression levels of immune-related genes. *Ladderlectin*, *GBP 1*, *Caspase-1*, *CLEC4F*, *MR1* and *GBP 2* were significantly down-regulated, but *HIF-1α*, *Cactus*, *TIPE*, *Akirin-2*, *Ivns1abp* and *TLR3* were significantly up-regulated. We further demonstrated that acute hypoxia activated *Yki* via *HIF-1α* to enhance expression level of *Cactus*, and then *Cactus* inhibited the phosphorylation of Dorsal and its nuclear translocation, thereby suppressing antibacterial immunity. Subsequently, the challenge experiment following stress revealed that exposure to acute hypoxia stress amplified the infectivity and lethality of *Vibrio parahaemolyticus* to shrimp. The mechanism of *HIF-1α-Yki-Cautus* axis provided an explanation for this phenomenon.

**Discussion:**

This study offered new insights into interactions among environmental hypoxia stress, host immunity and pathogens, thereby providing practical guidelines for optimizing shrimp culture practices.

## 1 Introduction

Hypoxia exerts significant impacts on the development, morphology, behavior, reproduction and physiological metabolism of aquatic animals (1–3). Hypoxia stress renders aquatic animals more susceptible to bacterial disease. The mortality of *Oreochromis niloticus* infected with *streptococcus agalactiae* was found to be significantly elevated under hypoxia condition (4), while the mortality of *Scylla paramamosain* infected with *Vibrio alginolyticus* also exhibited a notable increase (5). It is noteworthy that hypoxia has been shown to suppress the non-specific immune response of aquatic animals (6). For instance, exposure to hypoxia stress resulted in a significant decline in the activities of lysozyme and phenoloxidase in *Callinectes sapidus* and *Eriocheir sinensis* (7–9), while a notable decrease was observed in the haemolymph’s phagocytic, bacteriolytic, and antibacterial activities in *E. sinensis* and *M. rosenbergii* (10–12). Additionally, the exposure to hypoxia stress was found to significantly suppress the immune system of *Macrobrachium rosenbergii*, thereby augmenting its susceptibility to *Enterococcus* infection (13).

*Litopenaeus vannamei* is the shrimp species with the highest production in worldwide aquaculture due to its high growth rate and wide tolerance for salinity ranging between 0.5-40‰ (14). Its global production reached 6.32 million tons, which constitutes a staggering 51.7% of total shrimp output across the globe (15). Shrimp are the most sensitive to hypoxia stress in crustaceans (6). An adult *L. vannamei* consumes oxygen is about 1.23 mg.h^-1^, and their tolerance level is about 1.02 mg.L^-1^ (16). When water dissolved oxygen (DO) fell below the 3 mg.L^-1^, the phenoloxidase, phagocytosis, bacteriolytic and antibacterial activity in haemolymph of *L. vannamei* were remarkably suppressed (12). The suppression of these immune parameters increased its susceptibility to pathogen, such as the exposure of *L*. *vannamei* to cyclic serious/medium hypoxia resulted in an increased mortality upon challenge with *Vibrio parahaemolyticus* (17). *V. parahaemolyticus* is one of the most common bacterial pathogen that can widely infect fish, shellfish and crustacean (18), which harboring a virulence plasmid encoding *PirA/B* can cause a lethal disease in shrimp, known as the acute hepatopancreatic necrosis disease (AHPND) (19). This disease can result in a 100% mortality rate among infected shrimps within 30-35 days, leading to huge economic losses for the shrimp farming industry (20, 21). The hepatopancreas of the AHPND shrimp exhibit a pale yellow or light yellow coloration, accompanied by evident atrophy, and black spots or streaks become observable in advanced stages of the disease (20, 22). The intestines of the diseased shrimp exhibit emptiness, with a thinned midgut wall and detached intestinal mucosa floating in the lumen (20, 22). The progression of AHPND in *L*. *vannamei* is intricate and contingent upon a multitude of factors, including environmental, host-related, and pathogenic factors (18). The outbreak of bacterial diseases in shrimp is more likely to occur when anoxia and concentrations of ammonia and nitrite are excessively high in aquaculture (23). Most of the studies lack the mechanisms that elucidate the complex interplay between the environmental conditions and development of bacterial diseases for shrimp. Therefore, it is imperative to elucidate the underlying mechanism of bacterial disease outbreaks of shrimp in response to hypoxia stress.

*HIF-1*, composed of *HIF-1*α and *HIF-1*β, functions as a pivotal regulator of oxygen homeostasis (24). The role of *HIF-1α* in regulating host glucose metabolism has been extensively investigated in shrimp (6, 25), while limited information exists regarding the regulation of shrimp immune response by *HIF-1α*. Invertebrates including shrimp solely rely on innate immunity for their defense response, due to the absence of specific immunity based on immunoglobulin (26). The Toll-like receptors (TLR) is a pivotal regulator of the innate immune response in shrimp (27). TLR signaling induces the upregulation of diverse antimicrobial peptides through activation of the NF-κB transcription factor Dorsal, thereby exerting the protective function against bacterial infections (26). The Iκ‐B protein Cactus acts as a suppressor of Dorsal. Cactus impairs the phosphorylation of Dorsal and hinders Dorsal entry into the nucleus, thus impacting the antimicrobial capacity of hosts (26). The invertebrate *Yorkie* (*Yki*) is the homolog of mammalian *YAP*, which is a crucial effector within Hippo signaling pathway (28, 29). The pivotal role of Hippo signaling pathway in innate immunity of invertebrate is initially documented in 2016, wherein *Yki* directly facilitates the transcriptional activation of the *IκB* homolog *Cactus* in *Drosophila*, thereby repressing the expression of TLR-induced antimicrobial peptide genes (30). Thus, the inhibition of *Yki* augments host’s antibacterial immune response (30). *Yki* has been shown to inhibit the activation of *Dorsal* by up-regulating the *Cactus* in both *L. vannamei* and *Macrobrachium nipponense* (31–33). However, the interaction network of these pivotal genes in shrimp exposed to hypoxia stress remains unclear.

Herein, the intricate relationship among hypoxia, *V. parahaemolyticus* and host immunity in *L. vannamei* were studied. We conducted an acute hypoxia stress experiment to explore the impact of stress on immune response of host through transcriptome analysis and RT-qPCR. Then, we performed an injection challenge experiment with *V. parahaemolyticus* following stress to examine its colonization in the hepatopancreas of shrimp post-stress. Our findings revealed detrimental impact of acute hypoxia stress on attenuate antibacterial immunity of shrimp, rendering the host more susceptible to bacterial pathogen. Specifically, acute hypoxia stress induced *HIF-1α*-*Yki*-*Cactus* axis to inhibit the antibacterial immunity that weakens the shrimp’s resistance against *V. parahaemolyticus* infections.

## 2 Materials and methods

### 2.1 Experimental design and sample collection

**Figure 1:**
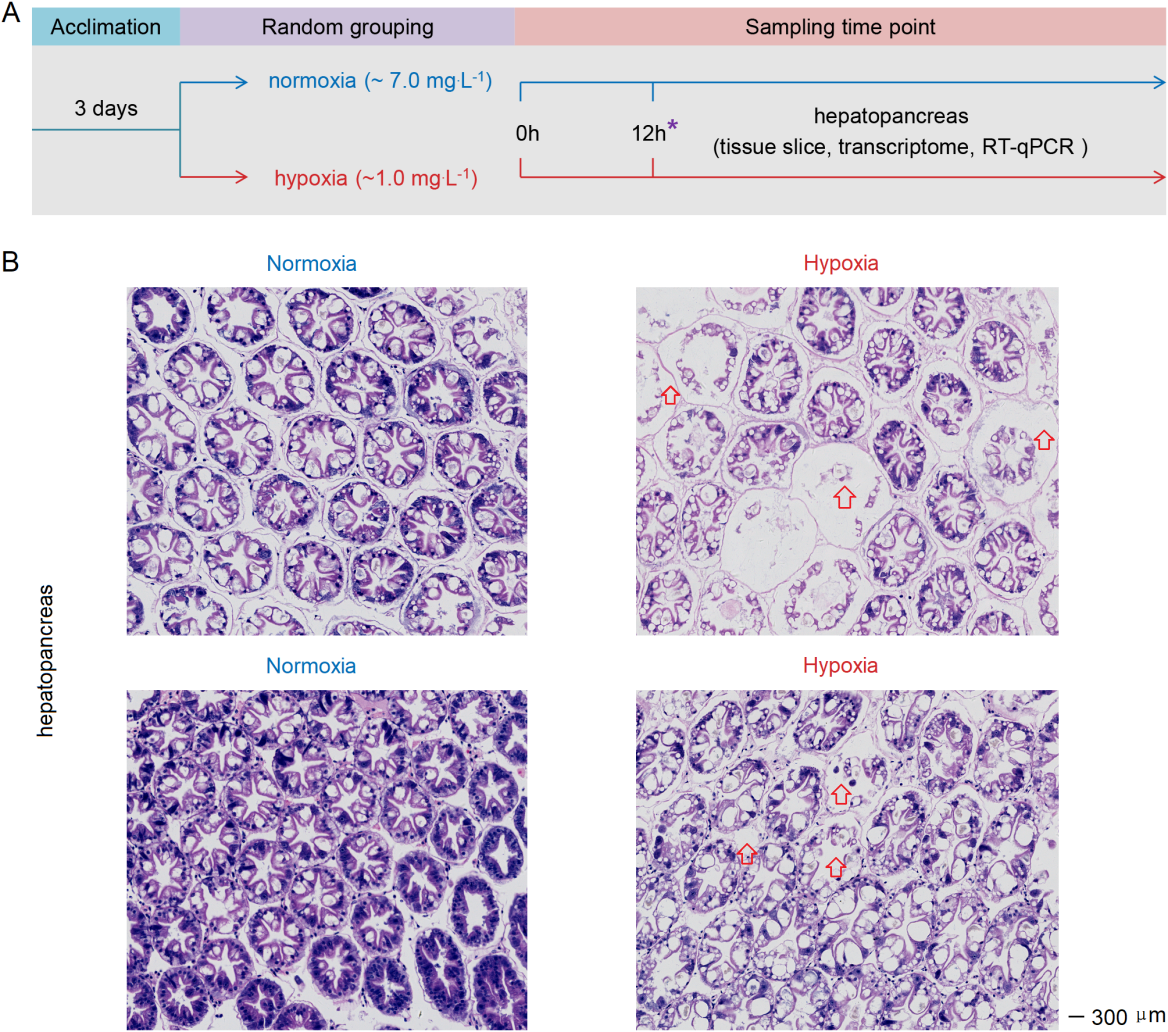
**(A)** Schematic representation of the experimental procedure for inducing acute hypoxia stress. **(B)** Acute hypoxia stress disrupted the tissue architecture in the hepatopancreas of shrimp. The arrow indicates severe atrophy of the glandular duct in the hepatopancreas of hypoxia-stressed shrimp, with the presence of gas bubbles observed between the glandular ducts. The symbol “*” represents the sampling time point.

*L. vannamei* (~ 6 g) were obtained from the breeding base of Doumen Nanhai Institute of Oceanology in Zhuhai, China. The salinity range of aquaculture ponds feeding *L. vannamei* in Zhuhai varies from 0.67 to 2.81‰ (34). Before the experiment, the shrimp were reared in a temporary tank with water temperature of 27°C and a salinity of about 3‰, and one-third of the water was replaced daily. Then, we conducted a hypoxia stress experiment (**Figure 1A**): Initially, shrimp were transferred from ponds to laboratory for temporary rearing. After two days, the shrimps were allocated into the hypoxia group (with controlled DO levels at approximately 1 mg.L^-1^) and normoxia group (approximately 7 mg.L^-1^) (**Supplementary Table S1**). Each group had three aquariums with 30 shrimp in each aquarium. We collected hepatopancreas from six randomly selected shrimp in each group at 3h, 6h and 12h post-stress. The tissue of one shrimp was classified as a separate sample. Samples were stored at -80°C prior to RNA extraction. Hepatopancreas were collected at 12h post-stress and subsequently processed into paraffin sections using the methodology described in previous study (35). The sections were stained using a Hematoxylin and Eosin Staining Kit (Beyotime; cat. no. C0105S), followed by microscopic examination.

### 2.2 The transcriptome sequencings

Total RNA was extracted using Trizol reagent, and the quantity and quality of RNA were assessed with a Nanodrop2000 spectrophotometer (Thermo Fisher Scientific, Waltham, MA, USA) and Agient2100 (Perkin Elmer, Waltham, MA, USA). RNA quality met the requirements for library construction as follows: The total amount ≥ 2 μg, concentration ≥ 40 ng.μL^-1^, volume ≥ 10 μL, OD260/280 ratio from 1.7 to 2.5, OD260/230 ratio from 0.5 to 2.5, normal display of the absorption peak at 260 nm, and RIN value ≥ 7. The qualified samples were enriched with eukaryotic mRNA by magnetic beads containing Oligo (dT). Then, mRNA fragmentation was achieved through random interruption using Fragmentation Buffer. First cDNA strand synthesis and subsequent double-stranded cDNA synthesis were carried out utilizing mRNA as a template, followed by purification of synthesized cDNA. cDNA underwent end-repair, a-tailing, and connection of sequencing adapters to form sequencing libraries with selected fragment sizes using AMPure XP beads. Finally, PCR enrichment yielded the desired cDNA library. After the construction of library, preliminary quantification was performed using Qubit 3.0 fluorometric quantifier at a concentration above 1 ng.μL^-1^, followed by the detection of inserted fragments using Qsep400 high-throughput analysis system. Once inserted fragments met expected criteria, accurate quantification of library’s effective concentration (> 2 nM) was conducted using RT-qPCR to ensure its quality. The cDNA library was subjected to sequencing on Illumina NovaSeq 6000 platform (Illumina, San Diego, CA, USA) at Biomarker Technologies Co. Ltd. (Beijing, China).

### 2.3 Bioinformatic analysis

We conducted raw data trimming using Trimmomatic (version 0.39) with default parameters and check quality using FastQC (version 0.11.8). Then, each set of clean reads was independently aligned to the *L*. *vannamei* genome (ASM378908v1) in orientation mode using TopHat. The expression level of each transcript calculated based on the fragment per kilobase of exon per million mapped reads (FPKM) method. The DESeq2 was employed to identify differentially expressed genes (DEGs) based on gene count values. During the process of differential expression analysis, screening criteria were set as false discovery rate (FDR) < 0.01, where fold change (FC) represented the ratio of expression levels between the normoxia and hypoxia groups and FDR was obtained by correcting the significance of differences with p-values. To facilitate comparison, differential FC was presented as log_2_FC. A larger absolute value of log_2_FC indicated a smaller FDR value for genes in two groups, highlighting more pronounced differences. The GO functional enrichment of DEGs was analyzed using Goatools, and KEGG pathway analysis of the DEGs was performed using KOBAS.

### 2.4 Experiment of *V*. *parahaemolyticus* injection challenge after stress and quantification of *V*. *parahaemolyticus*

We conducted an injection challenge experiment with *V*. *parahaemolyticus* following stress to investigate its colonization in hepatopancreas of shrimp post-stress. The *V. parahaemolyticus* originated from Guangdong Microbial Culture Collection Center, and harbor pVA1-like plasmids containing virulence genes pirA^Vp^ and pirB^Vp^. Shrimp (~6 g) were transferred to laboratory for temporary rearing. After two days, the shrimps were allocated into four groups: the hypoxia (whose DO was about 1 mg.L^-1^), hypoxia+*V*. *parahaemolyticus* (1 mg.L^-1^), normoxia (7 mg.L^-1^) and normoxia+*V*. *parahaemolyticus* (7 mg.L^-1^) groups (**Supplementary Table S1**). Each group had three aquariums with 30 shrimp in each aquarium. The previously preserved *V. parahaemolyticus* was inoculated into Luria-Broth medium at a dilution ratio of 1:100. The bacteria solution was incubated in a shaker at 180 r/min at 28°C for 14h, and then diluted into PBS at a concentration of about 10^5^ CFU per 50 μL to perform an injection challenge experiment when hypoxia stress reached 12h. The mortality rates in each group were recorded at 24h intervals for a duration of 168h following challenge, enabling the calculation of survival rates. Repeated the above experiment and collected the hepatopancreas from six randomly selected shrimp in each group at 6h, 12h and 24h following challenge. The tissue of each shrimp was classified as a sample. All samples were stored at -80°C before RNA and DNA extraction.

Extracted DNA from hepatopancreas (0.1 g) was subjected to absolute qPCR for quantification of *V*. *parahaemolyticus* (36), using primers listed in **Supplementary Table S2**. The plasmid pGEM-T-Easy containing a 336-bp DNA fragment from the pirA^Vp^ was serially diluted to 10-fold for the generation of a standard curve. The quantification of *V. parahaemolyticus* copies in the sample was determined based on the established standard curve. The PCR reaction mixture (10 μl) contained 5 μl LightCycler^®^480 Probes Master (Roche; cat. no. 04707494001), 2.5 μl DNA template, 1 μl of 10 μM primers and 0.02 μl of 100 μM TaqMan fluorogenic probe, 1.48 μl H_2_O. The absolute qPCR conditions were 1 cycle at 95°C for 8 min, 40 cycles of 10 s at 95°C, 25 s at 60°C, and 1 s at 72°C. Each sample was carried out with three replicates.

### 2.5 Immune-related genes quantification by RT-qPCR

Relevant tissues of shrimp were subjected to total RNA extraction using the Eastep Super Total RNA Extraction Kit (Promega, Shanghai, China). The genomic DNA of shrimp tissues was extracted using a genomic DNA extraction kit (V, Guangzhou, China). First-strand cDNA synthesis was performed using a cDNA synthesis kit (Takara, Dalian, China). The expression levels of genes were quantified using LightCycler 480 System (Roche, Basel, Germany) in a final reaction volume of 10 μL. This reaction mixture consisted of 1 μL of 1:10 cDNA diluted with ddH2O, 5 μL of GoTaq RT-qPCR Master Mix (Promega, Madison, WI, USA; cat. no. A6002), and 250 nM of specific primers. The primers utilized in this study are listed in **Supplementary Table S3**. The cycling program was conducted as follows: an initial cycle at 95°C for 2 min, followed by 40 cycles consisting of denaturation at 95°C for 15 s, annealing at 62°C for 1 min, and extension at 70°C for 1 s. The cycling process concluded with a final denaturation step at 95°C using a heating rate of 5°C/s to generate the melting curve. The expression level of each gene was determined using the Livak (2^-△△CT^) method after normalization to EF-1a (GenBank accession No. GU136229).

### 2.6 Detection of phosphorylation and nuclear migration of Dorsal

Hepatopancreas was collected from each group (n=5) at 12h post-stress and prepared for the extraction of total protein, nuclear protein and cytoplasmic protein, respectively. For total protein extraction, 30 mg hepatopancreas was treated with 200 μL of pre-added Protease Inhibitor Cocktail (Beyotime; cat. no. P1005) RIPA Lysis Buffer (Beyotime; cat. no. P0013B), thoroughly homogenized using a glass homogenizer, and subsequently incubated on ice for 30 min. The suspension was subjected to centrifugation at 15000 g and 4°C for 10 min. Obtained supernatant served as a total protein sample for assessing both the phosphorylation level of Dorsal and its overall protein abundance. Nuclear and cytoplasmic proteins were extracted from 30 mg hepatopancreas using a Nuclear and Cytoplasmic Protein Extraction Kit (Beyotime; cat. no. P0028), with the addition of Protease and Phosphatase Inhibitor Cocktail (Beyotime; cat. no. P1045) beforehand. Detailed steps can be found in the provided instructions. Protein samples were separated using SDS-PAGE gels. Then transferred to PVDF membranes (Merck; cat. no. IPVH00010) and incubated with the corresponding antibodies. Primary antibodies used in this study consisted of rabbit anti-Dorsal (Genecreate antibody customization), rabbit anti-p-Dorsal (Genecreate antibody customization), rabbit anti-β-actin (CST; cat. no. 8457T) and rabbit anti-PCNA (CST; cat. no. 13110T). The secondary antibody used in this study was anti-rabbit IgG HRP-conjugate (Promega; cat. no. W4011). These antibodies were diluted in TBS-T containing BSA (0.5%) for 2 h. The density of immunoblotted protein bands was quantified using ImageJ software. Same approach was employed to detect the phosphorylation and nuclear translocation of Dorsal in the hepatopancreas of *V*. *parahaemolyticus*-infected shrimp subjected to acute hypoxia stress.

### 2.7 Statistical analysis

Student’s *t*-test was used to compare differences in the expression levels of host genes detected by RT-qPCR and copy numbers of *V*. *parahaemolyticus* between the hypoxia and normoxia groups. The survival rates were analyzed using GraphPad Prism software to generate the Kaplan-Meier plot (log-rank χ2 test) for statistical analysis. Asterisks indicate statistical significance (* means *p* < 0.05; ** means *p* < 0.01), ns means no significance.

## 3 Results

### 3.1 Acute hypoxia stress damaged the hepatopancreas of shrimp

After 12h of hypoxia stress, the cellular connections within the hepatopancreas tissue of shrimp exhibited noticeable sparsity, accompanied by severe vacuolation and hepatic tubule destruction in the hepatopancreas cells (**Figure 1B**). Therefore, the acute hypoxia stress disrupted the tissue architecture in the hepatopancreas.

### 3.2 Acute hypoxia stress significantly changed the gene expression profiles

**Figure 2:**
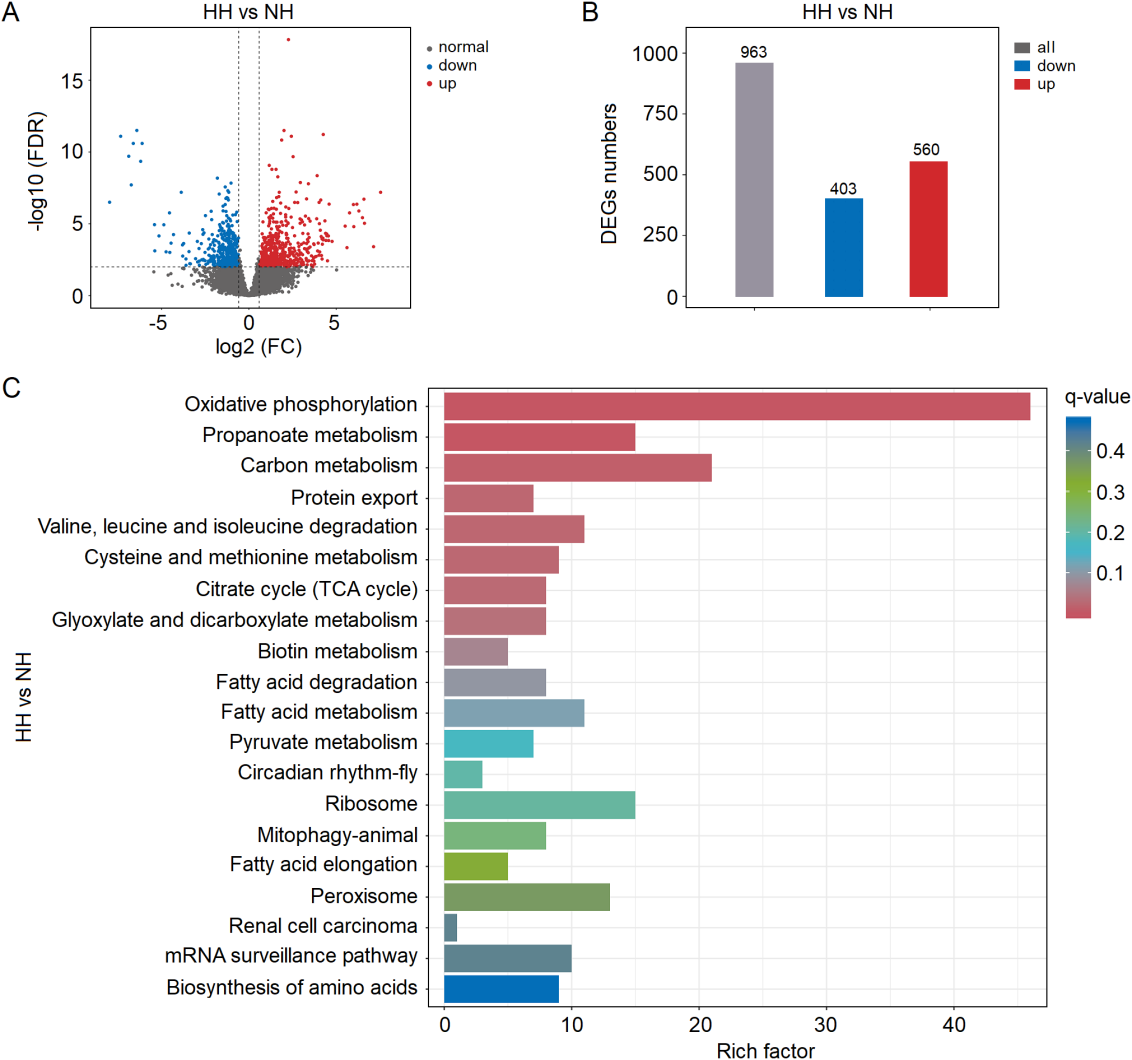
**(A)** Volcano plot analysis results of differentially expressed genes (DEGs) in the hepatopancreas of shrimp under acute hypoxia stress. **(B)** Numbers of DEGs in the hepatopancreas under stress. **(C)** KEGG annotation results of DEGs.

The Volcano plot analysis demonstrated significant (*P* < 0.05 in all cases) up- and down-regulation of numerous DEGs in the hepatopancreas under acute hypoxia stress (**Figure 2A**; **Supplementary Table S4**). There was a significant distinction in expression of differentially regulated genes between these two groups (**Supplementary Figure S1**). More precisely, a total of 963 DEGs were detected in the hepatopancreas tissue, with 410 being down-regulated and 560 up-regulated, respectively (**Figure 2B**). The KEGG annotation results revealed that the genes exhibiting significant alterations in the hepatopancreas tissue under hypoxia stress primarily contributed to oxidative phosphorylation (both down- and up-regulated), propanoate metabolism (both), carbon metabolism (both), protein export (only down), valine, leucine and isoleucine degradation (only down), cysteine and methionine metabolism (both), citrate cycle (TCA cycle) (only down), glyoxylate and dicarboxylate metabolism (only down), biotin metabolism (only down), fatty acid degradation (only down), fatty acid metabolism (both), pyruvate metabolism (both), circadian rhythm-fly (only up), ribosome (only down), mitophagy-animal (only up), fatty acid elongation (only down), peroxisome (only down), renal cell carcinoma (only up), mRNA surveillance pathway (both) and biosynthesis of amino acids (both) (**Figure 2C**; **Supplementary Figure S2**). Additionally, the GO annotation results revealed these DEGs were assigned to 33 GO_classify2 under three major categories (**Supplementary Figure S3**). The DEGs belonging to biochemical processes were mainly attributed to cellular process (337), metabolic process (275), biological regulation (148), response to stimulus (70), localization (97), signaling (44), developmental process (42), multicellular organismal process (37), multi-organism process (21) and reproduction (18). The DEGs belonging to cellular component were attributed to cellular anatomical entity (411), intracellular (224) and protein-containing complex (80), and which belonging to the molecular function were attributed to binding (294) and catalytic activity (275). All above results indicated that acute hypoxia stress significantly changed the gene expression profiles in the hepatopancreas of shrimp.

### 3.3 Acute hypoxia stress changed the expression of immune-related genes

**Figure 3:**
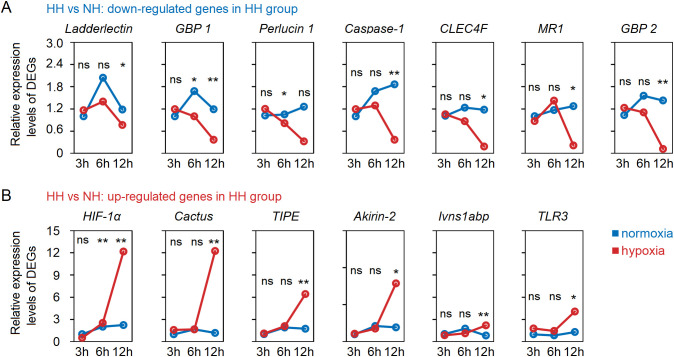
Dynamic changes of immune-related DEGs with down-regulated **(A)** and up-regulated **(B)** in the hepatopancreas tissues of shrimp under acute hypoxia stress. * means p < 0.05; ** means p < 0.01, ns means no significance.

Based on the gene annotation results, we performed NCBI sequence alignment for all DEGs, and identified 13 immune-related genes in the hepatopancreas, with 7 being down-regulated and 7 being up-regulated, respectively (**Supplementary Table S5**). The expression levels of LOC113813710 (*Ladderlectin*), LOC113813385 (*GBP 1*), LOC113809173 (*Perlucin 1*), LOC113823293 (*Caspase-1*), LOC113805525 (*CLEC4F*), LOC113819956 (*MR1*) and LOC113807239 (*GBP 2*) were significantly (*P* < 0.05 in all cases) down-regulated, while LOC113823783 (*HIF-1α*), LOC113823636 (*Cactus*), LOC113823947 (*TIPE*), LOC113823754 (*Akirin-2*), LOC113822408 (*Ivns1abp*), LOC113820234 (*TLR3*) and LOC113804912 (*Yki*) exhibited a significant up-regulation (**Supplementary Table S5**). We investigated the dynamic changes in expression levels of these 13 immune-related genes in the hepatopancreas under acute hypoxia stress (**Figure 3**). The results showed that *GBP 1* was significantly down-regulated from 6h, and the *Ladderlectin*, *Caspase-1*, *CLEC4F*, *MR1* and *GBP 2* were significantly down-regulated from 12h. *HIF-1α* was significantly up-regulated from 6h, and *Cactus*, *TIPE*, *Akirin-2*, *Ivns1abp* and *TLR3* were significantly up-regulated from 12h. More importantly, these data confirmed that *Ladderlectin*, *GBP 1*, *Caspase-1*, *CLEC4F*, *MR1* and *GBP 2* were significantly down-regulated, but *HIF-1α*, *Cactus*, *TIPE*, *Akirin-2*, *Ivns1abp* and *TLR3* were significantly up-regulated under stress at 12h.

### 3.4 Acute hypoxia stress mediated *HIF-1α-Yki-Cautus* axis to decrease immunity

**Figure 4:**
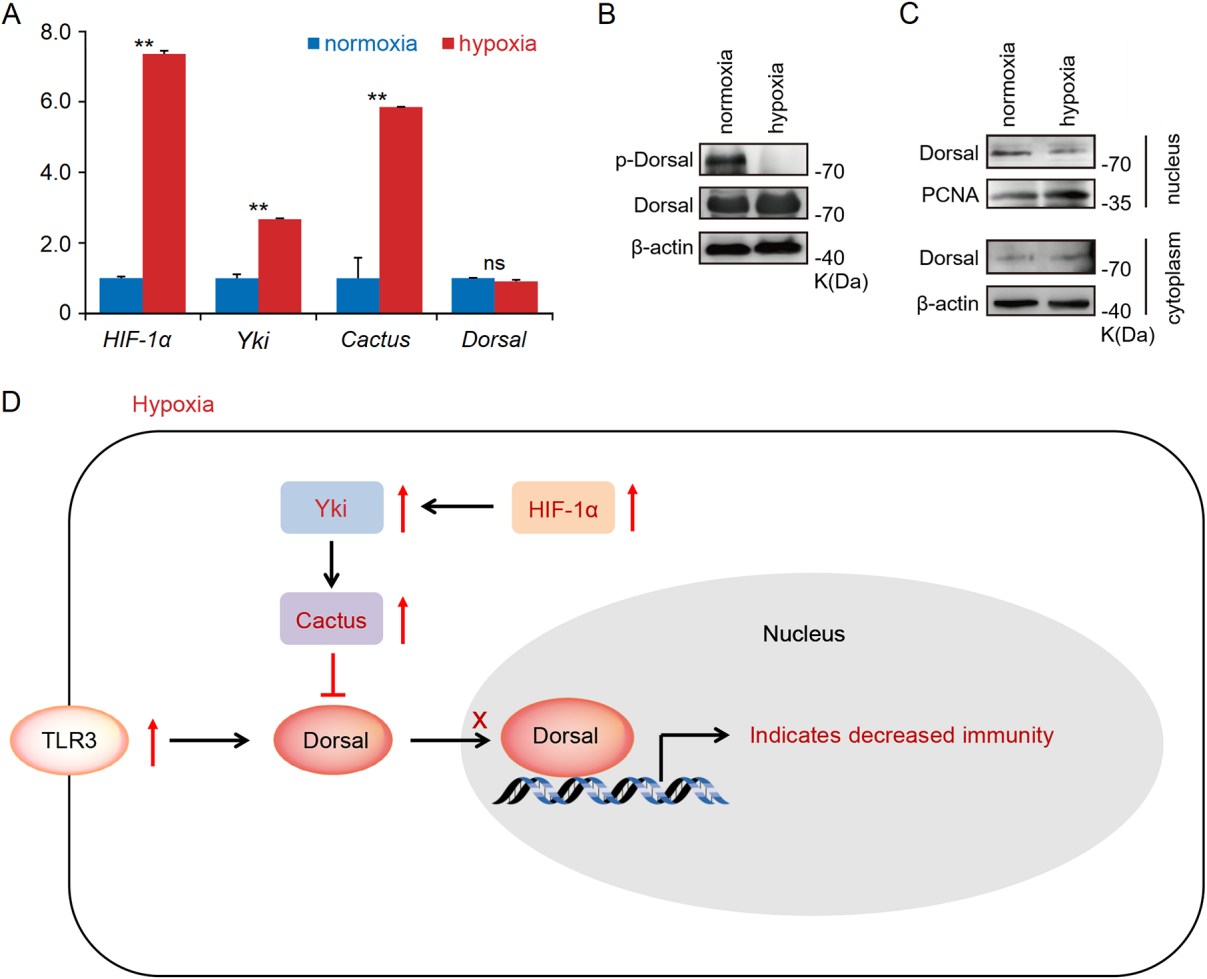
**(A)** The RT-qPCR results showed that a significant up-regulation of *TLR3* and *Cactus* expression levels in the hepatopancreatic under acute hypoxia stress, while expression level of *Dorsal* remained unaltered; concurrently, the expression levels of *HIF-1α* and *Yki* were found to be significantly up-regulated. **(B, C)** The WB results indicated that acute hypoxia stress inhibited the phosphorylation level of Dorsal in the hepatopancreas and reduced its nuclear translocation. **(D)** The expression of *HIF-1α* in the host was markedly up-regulated under acute hypoxia stress, subsequently inducing *Yki* expression, and this induction enhanced *Cactus*, and then *Cactus* ultimately impacted *Dorsal* entry into the nucleus. ** means p < 0.01, ns means no significance.

What is the underlying mechanism behind the substantial up-regulation of *Cactus* in response to acute hypoxia stress? Previous studies have demonstrated that the transcription factor *Yki* in Hippo signaling pathway promoted the expression level of *Cactus* (31, 37), while *HIF-1α* can directly induce the expression of *Yki* (38, 39). Indeed, our transcriptome and RT-qPCR results revealed a significant up-regulation of *TLR3* and *Cactus* expression levels in the hepatopancreas of shrimp subjected to acute hypoxia stress, while expression level of *Dorsal* remained unaltered; concurrently, the expression levels of *HIF-1α* and *Yki* (LOC113804912) were found to be significantly up-regulated (**Figure 4A**; **Supplementary Table S4**). Furthermore, our WB analysis revealed that acute hypoxia stress inhibited the phosphorylation level of Dorsal in the hepatopancreas and reduced its nuclear translocation (**Figures 4B, C**; **Supplementary Table S6**). Thus, although expression of *TRL3* was increased, then *Cactus* inhibited *Dorsal* entry into the nucleus. More significantly, the expression of *HIF-1α* in the host is markedly up-regulated under acute hypoxia stress, subsequently inducing *Yki* expression, and this induction enhances *Cactus*, and then *Cactus* ultimately impacted *Dorsal* entry into the cell nucleus, thereby influencing host immunity (**Figure 4D**).

### 3.5 Acute hypoxia stress facilitated the colonization of opportunistic pathogen in the hepatopancreas of shrimp

**Figure 5:**
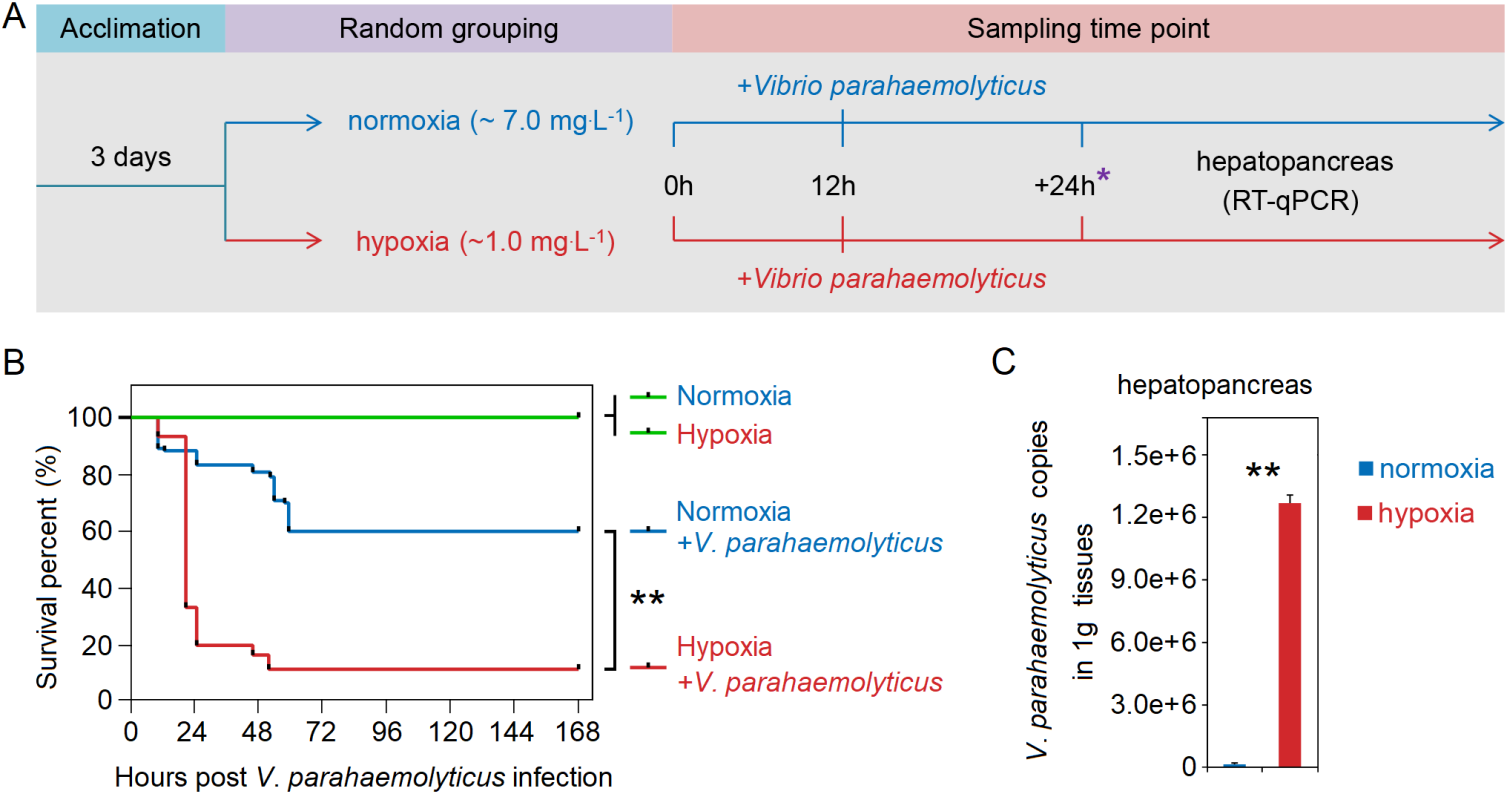
**(A)** Schematic representation of experimental procedure for an injection challenge experiment of *V. parahaemolyticus* subsequent to shrimp exposure to acute hypoxia stress. **(B)** Survival rate of shrimp in the hypoxia group was significantly lower compared to that in the normoxia group. **(C)** The copy numbers of *V*. *parahaemolyticus* in the hepatopancreas of shrimp in the hypoxia group were significantly higher than that in the normoxia group at 24h after challenge. * represents the sampling time point. ** means p < 0.01.

**Figure 6:**
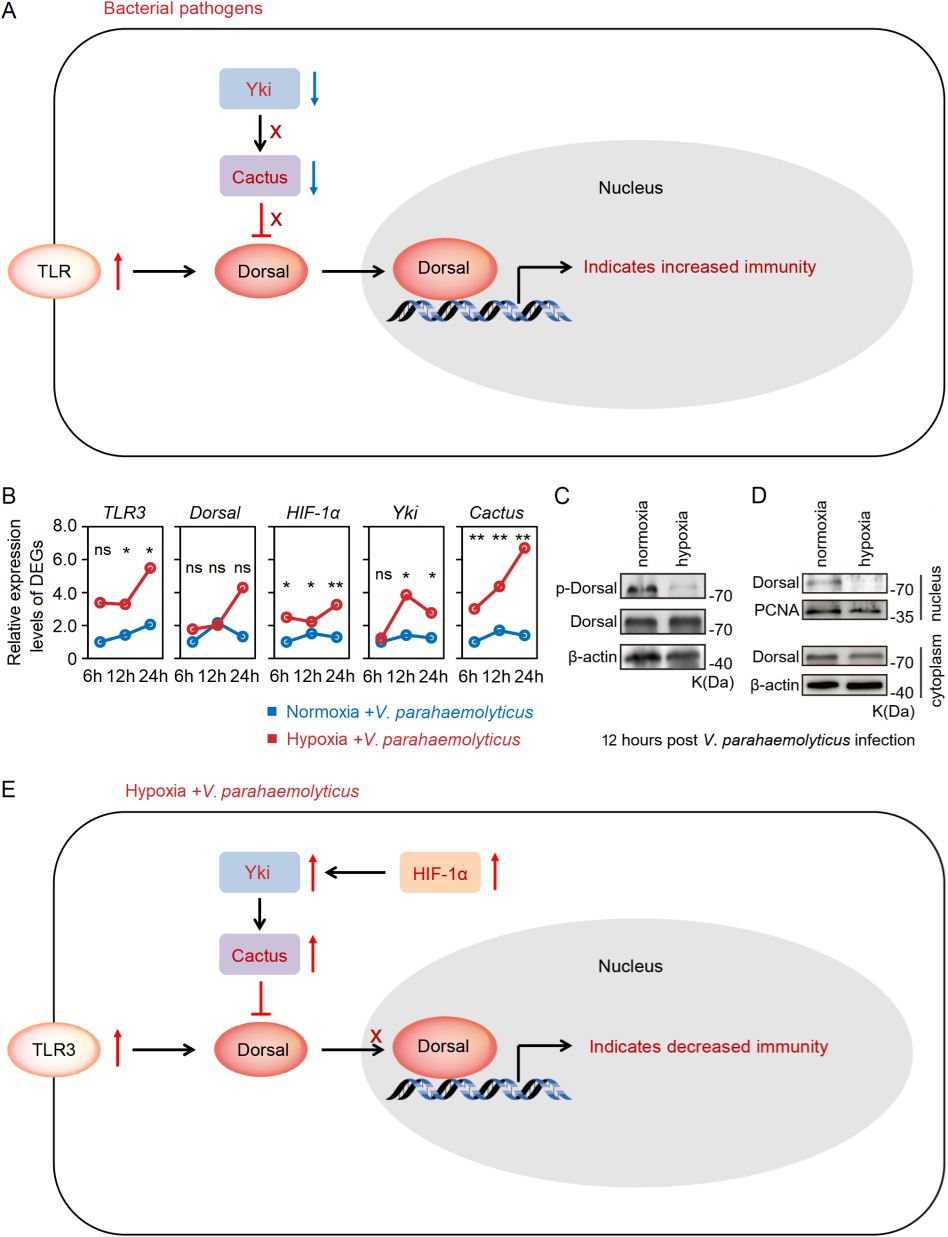
**(A)** Bacterial infection can rapidly activate *Hippo* through the regulation of toll-like receptors, resulting in inhibition of *Yki* and subsequent down-regulation of its downstream target gene *Cact*us, thereby triggering an immune response. **(B)** Acute hypoxia stress appeared to suppress immune capacity of host: the expression level of *TLR3* was significantly up-regulated with the challenge post-stress, but *HIF-1α* expression level was also markedly increased; consequently, there was a significant up-regulation of *Yki* and *Cactus* expression levels, leading to inhibition of *Dorsal*. **(C, D)** Acute hypoxia stress induced a reduction in Dorsal phosphorylation and inhibited its nuclear translocation within the hepatopancreas of shrimp after 12h of *V. parahaemolyticus* infection. **(E)** Acute hypoxia stress induced the *HIF-1α-Yki-Cactus* axis that promoted the *V*. *parahaemolyticus* infection to shrimp. * means p < 0.05; ** means p < 0.01, ns means no significance.

We conducted an injection challenge experiment of *V*. *parahaemolyticus* subsequent to shrimp exposure to acute hypoxia stress for 12h (**Figure 5A**). Survival rate of shrimp in both the normoxia and hypoxia groups reached 100% within 168h. However, the survival rate of hypoxia-stress shrimp was significantly lower compared to that of control shrimp, with only approximately 10% survival observed in the former and up to 60% in the latter (**Figure 5B**). A significantly (*P* < 0.05 in all cases) higher abundance of *V*. *parahaemolyticus* of shrimp in the hypoxia group at 24h after challenge, with the copies of *V*. *parahaemolyticus* in the normoxia group was approximately 8.8×10^3^ in the hepatopancreas, but it reached up to 1.3×10^6^ in the hypoxia group (**Figure 5C**). Under normal circumstances, bacterial infection can rapidly activate *Hippo* through the regulation of TLR, resulting in the inhibition of *Yki* and subsequent down-regulation of its downstream target gene *Cact*us, thereby triggering an immune response (30) (**Figure 6A**). However, we found that acute hypoxia stress appeared to suppress the immune capacity of host. Our results could explain this phenomenon: the expression level of *TLR3* was significantly up-regulated with challenge post-stress, but *HIF-1α* expression was also markedly increased; consequently, there was a significant up-regulation of *Yki* and *Cactus* expression levels, while there was no significant difference in the expression of *Dorsal* (**Figure 6B**). Importantly, acute hypoxia stress induced a reduction in Dorsal phosphorylation and inhibited its nuclear translocation within the hepatopancreas after 12h of *V. parahaemolyticus* infection (**Figures 6C, D**; **Supplementary Table S6**). Interestingly, *Yki* expression was initially not significantly changed at 6h with challenge post-stress; however, it was subsequently significantly up-regulated at 12h and 24h (**Figure 6B**). Acute hypoxia stress induces a significant up-regulation of *HIF-1α* and then which induces *YAP* (*Yki* homolog in mammal) (38, 39), while pathogen infection inhibits the expression of *Yki* (30). Our findings suggested that although pathogen infection temporarily inhibited *Yki* following acute hypoxia stress, it cannot overcome the induction effect of *HIF-1α* on *Yki*. Therefore, *Yki* expression was significantly up-regulated at 12h and 24h with challenge post-stress. Although both the hypoxia and *V*. *parahaemolyticus* infection could induce the up-regulation of *TLR3* expression, but *HIF-1α* up-regulation exerted a stronger induction effect on *Yki*; consequently, significantly increased-*Yki* expression promoted the up-regulation of *Cactus*, thereby inhibiting *Dorsal* entry into the nucleus (**Figure 6E**). Ultimately, this led to an increased infectivity of *V*. *parahaemolyticus* and a higher mortality in shrimp.

## 4 Discussion

The emergence of diseases is a consequence of interactions between host immunity, bacterial pathogens and environmental stress (40–42). This study elucidated the intricate interplay among acute hypoxia stress, *L*. *vannamei* and *V*. *parahaemolyticus*. Our study provided a molecular explanation for how acute hypoxia suppresses the host immunity and enhances susceptibility to bacterial infections.

Exposure to hypoxia has been demonstrated to suppress the non-specific immune response of aquatic animals (6), but molecular mechanisms underlying the immune defense response to hypoxia remain poorly understood. Our findings demonstrated that acute hypoxia stress diminished the resistance of *L. vannamei* by activating the *HIF-1α*-*Yki*-*Cactus* axis to suppress the role of *Dorsal* in antimicrobial response. This elucidation of the molecular mechanism, combined with previously reported biochemical and physiological parameters, provided insights into how hypoxia attenuates shrimp immune response. The expression of *HIF-1α* in the majority of decapods significantly increases under hypoxia stress, aligning with previously reported hypoxia responses observed in vertebrates (25). The relationship between hypoxia and metabolic genes targeted by *HIF-1α* has been investigated in several decapod crustaceans (6). In *L. vannamei*, the downstream genes of *HIF-1α* are involved in glucose metabolism (43–47). In mammals, *HIF-1α* and *YAP* were found to be co-activators of each other (48, 49). *HIF-1α* facilitates *YAP* activation by enhancing its expression and facilitating nuclear localization, thereby significantly promoting tumor cell proliferation, invasion, and differentiation (48, 50). On the other hand, hypoxia stress promotes the binding of *YAP* to *HIF-1*α in the nucleus, maintaining the stability of *HIF-1*α protein and participating in processes such as cartilage differentiation and glycolysis (51, 52). Therefore, a potential positive feedback regulation between *HIF-1*α and *Yki* may exist in response to hypoxia stress, and this regulation may be a conserved response to hypoxia. However, the regulatory mechanism between *HIF-1α* and *Yki* in shrimp remains poorly understood and necessitates further investigation. Our study unveiled the inhibitory impact of *HIF-1α* on *Dorsal*-induced antibacterial immune of hosts during acute hypoxia stress, thereby expanding our comprehension of *HIF-1α*’s immunological functions in shrimp. A recent study has reported that ammonia stress can induce increased *Cactus* expression through *heat shock factor 1*, resulting in inhibition of an arthropod interferon analog *Vago*-L produced by NF-κB pathway, ultimately facilitating infection by white spot syndrome virus (WSSV) in *M. japonicus* (53). It has also been reported that WSSV promotes *Yki* activation by inhibiting the Hippo signaling pathway, thereby inhibiting the *Dorsal* mediated anti-viral mechanism and promoting viral infection (54). These studies and our findings suggest that the *HIF-1α-Yki-Cactus* axis can influence the host immune response to pathogens, including bacteria and viruses, by modulating the Toll pathway. We suspected that *Cactus* may serve as a pivotal molecule involved in response to diverse stresses and act as a regulatory switch modulating host resistance against pathogens by regulating immune pathways. Further investigations are urgently needed to unravel intricate regulatory networks governing the immune gene responses in crustaceans exposed to hypoxia stress.

Additionally, our study unveiled significant alterations in the gene expressions under acute hypoxia stress, involved in the oxidative phosphorylation, propanoate metabolism, carbon metabolism in the hepatopancreas. Similarly, hypoxia stress upregulates the transcription of genes associated with glycolysis and the Krebs cycle pathways in *L. vannamei*, leading to an accumulation of carbohydrates and lactic acid (43). *M. rosenbergii* exhibits enhanced activity of glycolytic-related enzymes, reduced mRNA expression levels of aerobic respiratory enzymes, and significantly decreased concentrations of various amino acids under hypoxia condition (55). Notably, host metabolism plays a pivotal role in both environmental stress and pathogen infection. Bacterial pathogens can utilize the host cell metabolism via diverse mechanisms to promote their infection. *Salmonella* induces the metabolic reprogramming of macrophages through the T3SS effector SopE2 that enhancing cellular glycolysis, and the accumulated 3-phosphoglyceric acid serves as a carbon source to facilitate *Salmonella* replication, while the accumulated pyruvate and lactate acts as signaling molecules to activate the expression of *Salmonella* virulence factors (56). *Edwardsiella piscicida* utilizes the polyamine transport system of fish to metabolize host cytoplasmic arginine into ornithine, effectively impeding the efflux of host potassium ions and thereby inhibiting NLRP3 inflammasome activation, ultimately facilitating systemic infection by *E. piscicida* in host (57). Therefore, the exploitation of host metabolism by bacteria plays a pivotal role in their successful establishment of infection. We suspected that hypoxia stress may create a more conducive environment for pathogenic bacterial infections by modulating host metabolism, including glycolysis, fatty acid metabolism and amino acid metabolism, thereby facilitating the onset of bacterial diseases.

Our study also found that acute hypoxia stress rendered *L*. *vannamei* more susceptible to infection by *V*. *parahaemolyticus*, thereby leading to an increase in host morbidity and mortality. *Vibrio* genus exhibits a wide distribution in estuarine, coastal, and pelagic waters, sediments, as well as aquatic animals (58). Numerous species within this genus have been identified as potential pathogens (59, 60), such as the *V. parahemolyticus* (61), *V. harveyi* (62), *V. alginolyticus* (63), and *V. vulnificus* (64). Vibriosis has long been a persistent challenge in aquaculture, particularly in mariculture (65, 66). The pathogenic *Vibrio* can infect a wide range of host species, and its proliferation and dissemination are highly influenced by aquaculture environment (58, 67, 68). During the culturing process, the continuous influx of nutrients such as nitrogen and phosphorus promotes the proliferation of microorganisms like bacteria and algae (59, 61). Excessive biological oxygen demand or algal blooms can lead to oxygen depletion in aquaculture water (2). In such circumstances, if *Vibrio* pathogens are present, it can trigger epidemic and explosive death of economic animals such as farmed fish, shellfish and crustaceans (61). Given extensive distribution of *Vibrio* pathogens, the expansion of anoxic areas will inevitably augment the morbidity and mortality of bacteriosis among aquatic animals in aquatic ecosystems (69). Therefore, it is crucial to investigate the impact of vibriosis under hypoxia stress on the aquaculture and aquatic ecosystem.

**Figure 7:**
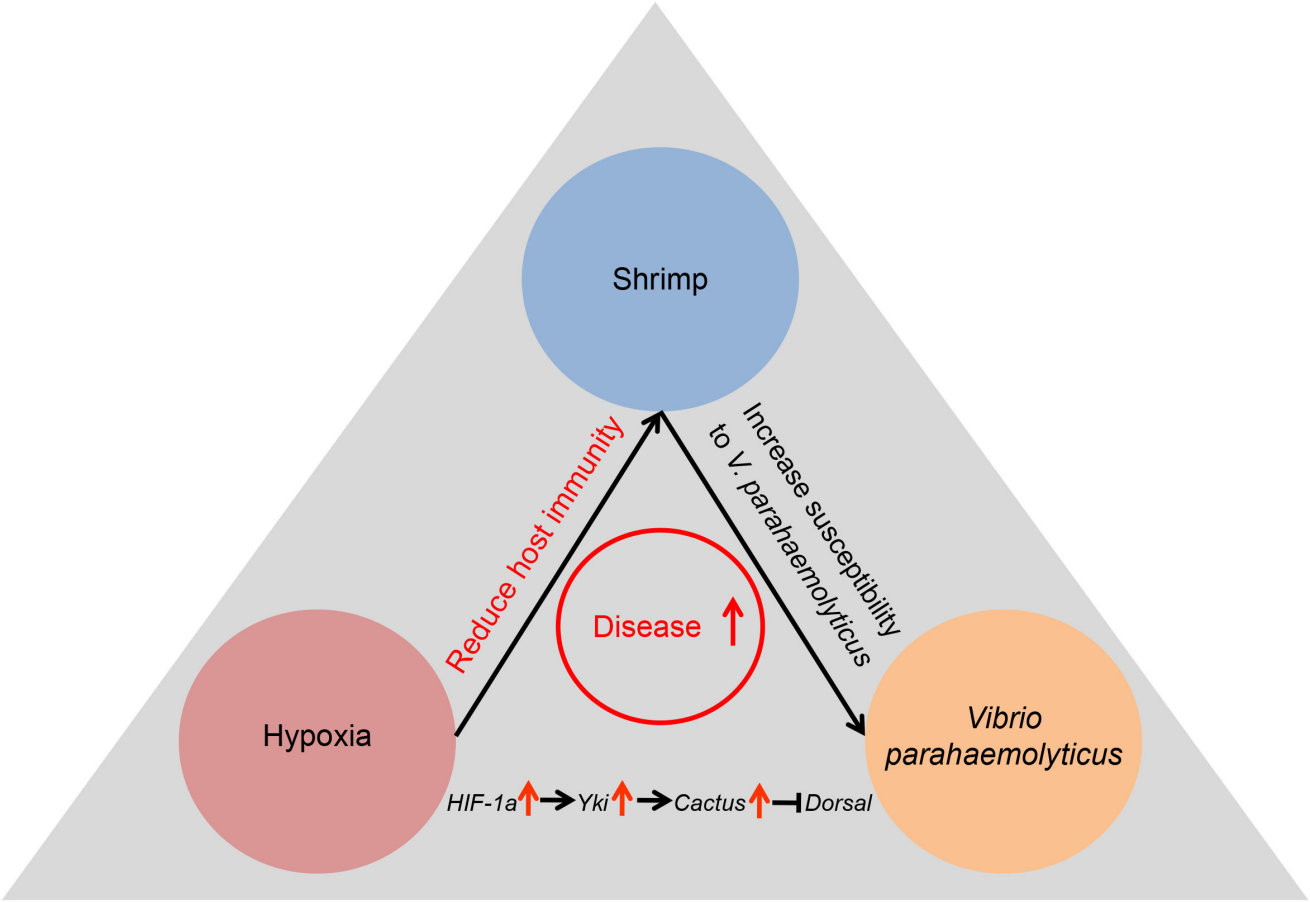
Acute hypoxia stress induced the tissue damage in digestive system of shrimp, compromising its immune response and rendering it susceptible to bacterial infection, ultimately resulting in the bacterial disease outbreaks.

In conclusion, this study demonstrated that acute hypoxia stress disrupted the tissue architecture and significantly changed genes expression profiles in the hepatopancreas of shrimp. More importantly, acute hypoxia stress suppressed the antibacterial immunity of shrimp by inhibiting the Dosal-induced signaling pathway through the *HIF-1α-Yki-Cactus* axis (**Figure 7**). This research provided novel insights into interactions among the hypoxia stress, host immunity and pathogen and established a theoretical foundation for strategies aimed at preventing and managing outbreaks of bacterial diseases in hypoxia environments.

## Data Availability

The datasets presented in this study can be found in online repositories. The names of the repository/repositories and accession number(s) can be found below: https://www.ncbi.nlm.nih.gov/, PRJNA1119264.
