# Supplementary material for: Acute hypoxia stress mediates HIF-1α-Yki-Cactus axis to facilitate the infection of Vibrio parahaemolyticus in Litopenaeus vannamei
Source: Front Immunol. 2024 Nov 27;15:1476309. doi: 10.3389/fimmu.2024.1476309 (PMC11632965; doi:10.3389/fimmu.2024.1476309)
Supplement: Supplementary file 1 [file DataSheet1.docx]

**Frontiers in Immunology**

**Supplementary Material for**

**Acute hypoxia stress mediates *HIF-1α-Yki-Cactus* axis to facilitate the infection of *Vibrio parahaemolyticus* in *Litopenaeus vannamei***

Honghui He^1,2^, Shaoqing Huang^3^, Ningze Geng^1^, Shaoping Weng^1,2^, Jianguo He^1,2^*, Chaozheng Li^1,2^*

^1^State Key Laboratory of Biocontrol/School of Marine Sciences, Sun Yat-sen University, Guangzhou, China/Southern Marine Sciences and Engineering Guangdong Laboratory (Zhuhai), Zhuhai, China

^2^School of Life Sciences, Sun Yat-sen University, Guangzhou, China/China-ASEAN Belt and Road Joint Laboratory on Mariculture Technology

^3^College of Marine Sciences, Beibu Gulf University, Qinzhou, China

*Corresponding Author

Chaozheng Li, lichzh5@mail.sysu.edu.cn

Jianguo He, Email: lsshjg@mail.sysu.edu.cn

This file contains the Supplementary Figures 1 to 3.

**
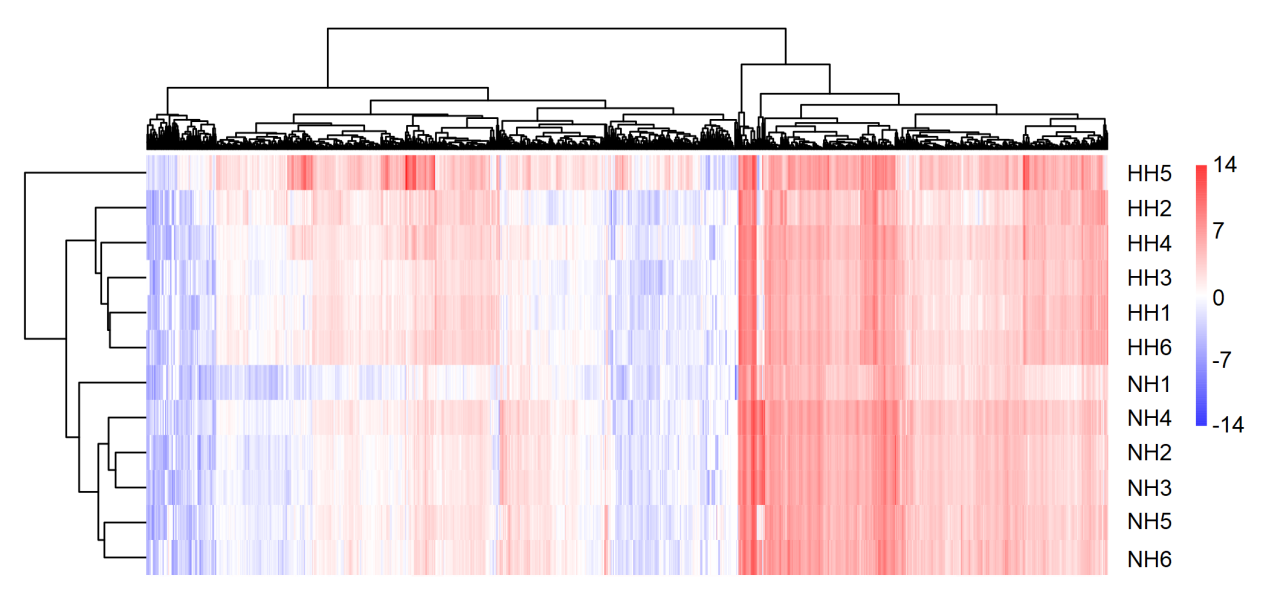
**

**FIGURE S1** There was a significant distinction in expression of differentially regulated genes between the normoxic and hypoxic groups in the hepatopancreas.


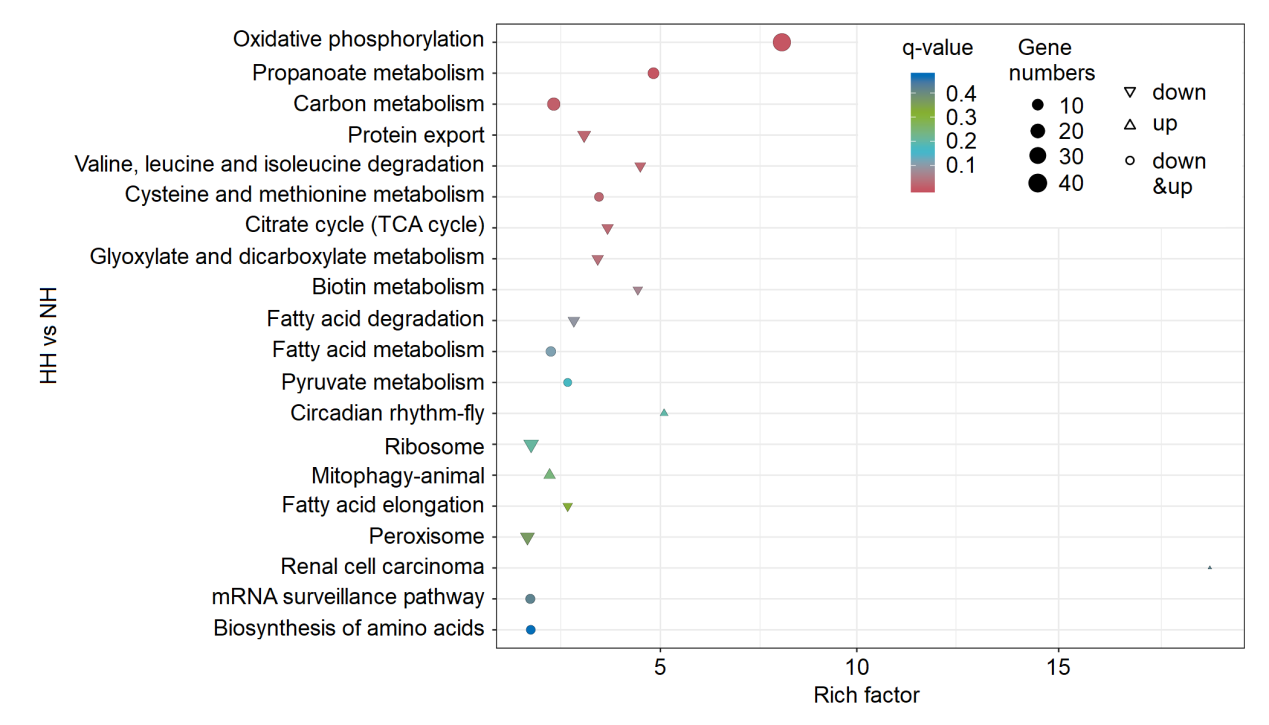


**FIGURE S2** The KEGG annotation results of down- and up-regulated DEGs in the hepatopancreas under acute hypoxic stress.


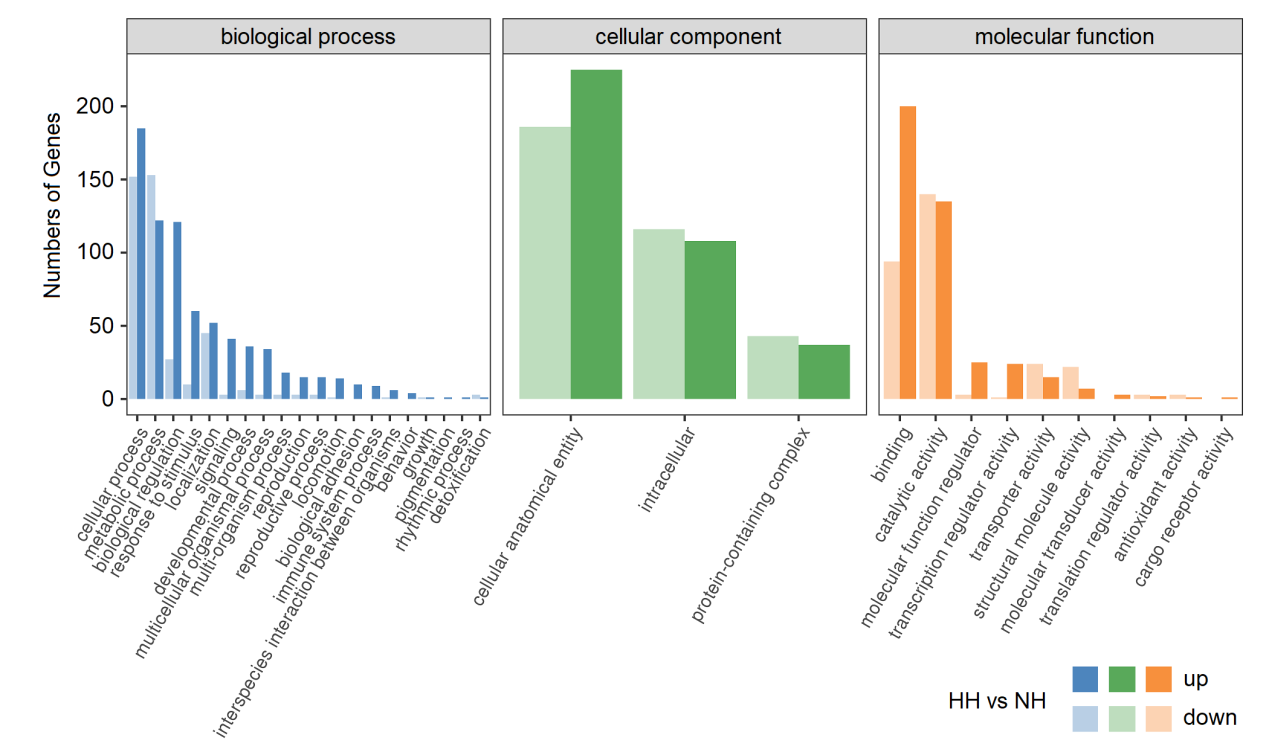


**FIGURE S3** The GO annotation results of DEGs in the hepatopancreas under acute hypoxic stress.
